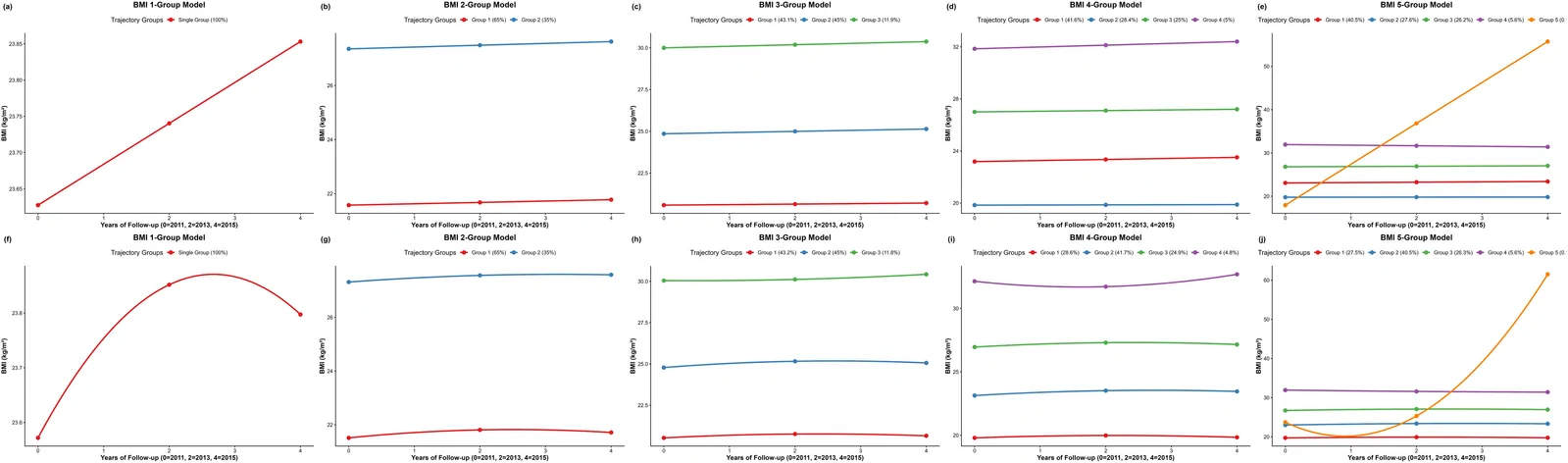# Mediating role of frailty in the association between systolic blood pressure trajectories and catastrophic health expenditure among middle-aged and older adults: A longitudinal cohort study

**DOI:** 10.1016/j.jnha.2026.101021

**Published:** 2026-10-30

**Authors:** Weili Liu, Zhonghua Yin, Ke Li

**Affiliations:** a Shifang People's Hospital, Shifang, China; b School of Life Sciences and Technology, University of Electronic Science and Technology of China, Chengdu, China

**Keywords:** Systolic blood pressure, Trajectories, Frailty, Catastrophic health expenditure, Mediation analysis, Middle-aged and older adults

## Abstract

**Background:**

Routine hypertension management primarily targets acute cardiovascular events, frequently overlooking progressive physiological decline (frailty) and its economic implications. This study aimed to identify systolic blood pressure (SBP) trajectories and evaluate the mediating role of frailty in their association with catastrophic health expenditure (CHE) among middle-aged and older adults.

**Methods:**

We analyzed 3996 participants from the China Health and Retirement Longitudinal Study (CHARLS). Group-based trajectory modeling identified SBP evolution patterns (2011–2015). The 2015 Frailty Index (FI), based on a 27-item deficit accumulation model, served as the mediator. The primary outcome was CHE in 2018, defined as annual household healthcare expenditure exceeding 40% of the household’s capacity to pay. Mediation analysis quantified total, direct, and indirect effects.

**Results:**

Three SBP trajectories emerged: “Low-stable” (50.0%), “Moderate-increasing” (41.3%), and “High-decreasing” (8.7%). Compared with the Low-stable group, the High-decreasing trajectory was significantly associated with higher 2015 FI scores (*β* = 0.029, *P* < 0.001). A statistically significant indirect effect through frailty was observed for the association between the High-decreasing trajectory and CHE in 2018 (ACME = 0.0084, 95% CI: 0.0031–0.0100, *P* < 0.001), although the direct and total effects were not statistically significant. Findings were similar in sensitivity analyses using alternative CHE definitions and BMI trajectories.

**Conclusions:**

Frailty may be one pathway linking the High-decreasing SBP trajectory with subsequent household CHE. These observational findings support considering functional health alongside blood pressure in middle-aged and older adults. Further prospective studies are needed to clarify the temporal and causal relationships and to assess whether incorporating frailty assessments into cardiovascular management improves clinical and socioeconomic outcomes.

## 1 Introduction

Hypertension remains a leading global public health challenge. As of 2024, it affects 1.4 billion individuals worldwide [1], with its prevalence in China increasing to 31.6% over the past decade [2]. As the primary modifiable cardiovascular risk factor, hypertension causes severe clinical complications and imposes a substantial economic burden, equivalent to 1% of China's gross domestic product (GDP) [3]. In China, CHE has been reported in 8.9% of households, while 4.1% of households have experienced medical impoverishment [4]. However, the pathophysiological damage caused by hypertension is a chronic, cumulative process. Current risk assessments rely heavily on single-timepoint blood pressure measurements, thereby limiting the accurate evaluation of true long-term exposure and subsequent health risks.

Blood pressure (BP) trajectories integrate multiple measurements over time, capturing both absolute levels and long-term trends [5,6], and may improve the assessment of subsequent health risks [7]. Trajectory analyses have identified patterns associated with subclinical target-organ damage, including coronary artery calcification and left ventricular hypertrophy [5,8,9]. They have also been used to characterize BP patterns in populations such as people with diabetes, pregnant women, and adolescents [[10], [11], [12]]. In addition, long-term BP dysregulation has been associated with increased healthcare utilization [13] and greater financial strain on households [14].

Existing health economic evaluations of hypertension predominantly focus on acute events like stroke [13], overlooking the progressive depletion of systemic physiological reserve. The Frailty Index (FI) provides a standardized framework to quantify this decline [15]. Evidence suggests that long-term BP dysregulation may be associated with multisystem health-deficit accumulation and an increased risk of frailty, potentially through pathways involving microvascular dysfunction and metabolic disturbances [[16], [17], [18], [19], [20], [21], [22]]. Frailty, in turn, is associated with functional dependency, recurrent hospitalizations, and greater healthcare costs. Among older adults, greater frailty has been associated with higher healthcare and out-of-pocket expenditures [[23], [24], [25], [26]], with a potentially greater burden among those with cardiovascular conditions [27].

Collectively, these findings suggest a plausible pathway in which long-term BP dysregulation may be associated with greater physiological deficit accumulation and frailty, which may, in turn, be associated with higher household healthcare spending and CHE. However, this potential pathway has not been fully examined among middle-aged and older adults. Accordingly, using nationally representative data from CHARLS, we aimed to characterize SBP trajectories from 2011 to 2015 and examine their association with CHE in 2018. We also assessed whether frailty measured in 2015 statistically mediated this association.

## 2 Methods

### 2.1 Study population

Data for this study were derived from the China Health and Retirement Longitudinal Study (CHARLS), a nationally representative prospective longitudinal cohort study. The baseline survey (2011–2012) employed a multi-stage stratified probability-proportional-to-size (PPS) sampling design, covering the middle-aged and older population across 150 county-level units nationwide. The study protocol was approved by the Biomedical Ethics Review Committee of Peking University, and written informed consent was obtained from all participants [28].

To preclude the anomalous disruptions of the 2020 coronavirus disease 2019 (COVID-19) pandemic on individuals' routine healthcare-seeking behaviors and health economic expenditures, this study utilized continuous follow-up data from the CHARLS baseline (2011) to the fourth wave (2018).

The detailed sample selection process for this study is illustrated in Fig. 1. The initial cohort comprised 16,903 baseline respondents aged > 45 years. To satisfy the temporal precedence assumption for causal inference, individuals who self-reported a history of severe cardiovascular and cerebrovascular diseases (e.g., stroke, myocardial infarction), heart failure, or malignancies were a priori excluded (remaining *n* = 14,462). To meet the requirements for trajectory modeling, participants lacking complete SBP measurements across the three time points between 2011 and 2015 were subsequently excluded (remaining *n* = 6196). Among the remaining respondents, those with missing data precluding the calculation of the Frailty Index (FI) or lacking body mass index (BMI) data (remaining *n* = 4997), lacking annual financial expenditure information (remaining *n* = 4078), or having incomplete covariate records were sequentially excluded. Ultimately, a total of 3996 participants were included in the present study. To evaluate potential selection bias, baseline characteristics were compared between the included (*N* = 3996) and excluded (*N* = 10,466) participants (Supplementary Table 1). The comparison revealed that while excluded individuals were generally older and had higher baseline SBP—indicating a 'healthier survivor effect' in our final cohort—the prevalence of CHE remained comparable between the groups.

**Fig. 1:**
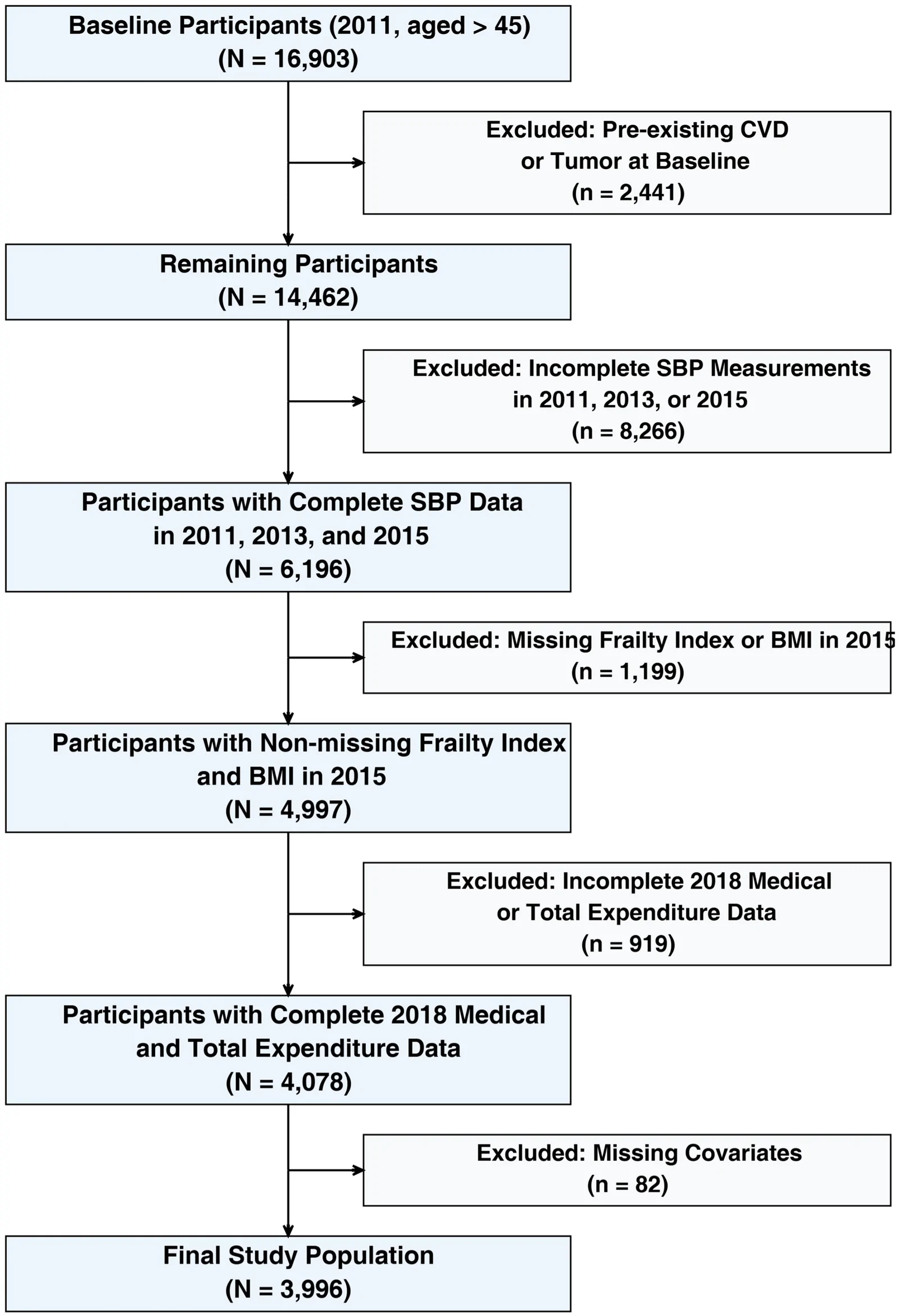
Flow of participants in current CHARLS study.

### 2.2 Blood pressure measurement and trajectory construction

Blood pressure (BP) measurements adhered to the standardized operating procedures of CHARLS. After participants rested in a seated position for 5 min, their left-arm BP was measured by professionally trained interviewers using an automated electronic blood pressure monitor (Omron HEM-7200). Three consecutive measurements were taken during each interview with a 45-second interval between readings, and the average of these three readings was defined as the BP value for that specific wave. To capture the dynamic evolution of long-term BP workload, this study extracted longitudinal systolic blood pressure (SBP) data across three time points (2011, 2013, and 2015), which served as the primary exposure variable for subsequent group-based trajectory modeling.

### 2.3 Frailty assessment

Conceptually, the SBP trajectory derived from 2011 to 2015 captures an inherent, latent phenotype of longitudinal blood pressure evolution. To explore the progressive physiological degradation driven by this latent phenotype, the year 2015 was designated as the intermediate snapshot for evaluating frailty status, fulfilling the mechanistic requirement of capturing cumulative functional decline prior to the onset of the ultimate economic outcome in 2018. The Frailty Index (FI), formulated based on the deficit accumulation model, was utilized to quantify the magnitude of physiological decline in individuals [29]. Drawing upon previously standardized protocols [[30], [31], [32]], this study incorporated 27 age-related health deficit items spanning three primary dimensions: (1) physical function and disability; (2) clinical symptoms and chronic diseases; and (3) psychological and cognitive function. Dichotomous items were assigned a score of 1 (deficit present) or 0 (deficit absent), whereas continuous variables (e.g., cognitive scores) were transformed onto a 0–1 scale using min-max normalization. For each participant, the FI was calculated using the following formula:

$$FI=\frac{\sum_{i=1}^{n}\chi_{i}}{n}$$

Where $\chi_{i}$ represents the score of the i-th deficit, and n denotes the total number of deficits measured.

The FI is a continuous variable ranging from 0 to 1, where higher scores indicate more severe functional deterioration. Based on widely recognized cut-off values, participants were stratified into three categories: robust (FI ≤ 0.10), pre-frail (0.10 < FI < 0.25), and frail (FI ≥ 0.25). The specific 27 constituent items of the FI [30] and their detailed definitions are delineated in Supplementary Table 2.

### 2.4 Healthcare expenditure and economic burden indicators

This study constructed the primary economic burden indicators utilizing the household income and expenditure module from the 2018 wave of CHARLS. To ensure comparability across varying statistical metrics, all consumption items with different recall periods (i.e., weekly, monthly, or annually) were uniformly converted into annualized values. Specifically, total annual household expenditure encompassed the aggregated expenses on food, tobacco, alcohol, daily necessities, and durable goods and services. Annual healthcare expenditure comprised direct out-of-pocket (OOP) medical expenses incurred by the household over the past year, as well as indirect costs associated with seeking healthcare, such as transportation, nutritional supplements, and caregiving fees. On this basis, the healthcare expenditure share—defined as the proportion of healthcare expenditure relative to the total annual household expenditure—was employed to measure household financial strain. Furthermore, this study defined catastrophic health expenditure (CHE) as a key dichotomous variable. In accordance with internationally recognized evaluation frameworks, a household was identified as having experienced CHE when its annual healthcare expenditure exceeded 40% of its capacity to pay (defined as total annual household expenditure minus food-related expenses) [33,34]. The detailed constituent components of each variable are delineated in Supplementary Table 3.

### 2.5 Covariates

To adjust for baseline confounding, socio-demographic, lifestyle, and clinical variables from the 2011 survey were incorporated into the models. Socio-demographic characteristics encompassed age, sex, residential area (urban or rural), educational attainment, and marital status. Lifestyle behaviors included smoking status and alcohol consumption history. Clinical variables included self-reported dyslipidemia and diabetes or high blood sugar. All covariates were extracted exclusively from the 2011 baseline wave to ensure the temporal sequence of the analysis.

### 2.6 Statistical analysis

Group-based trajectory modeling (GBTM) was employed to identify the heterogeneous evolution patterns of SBP from 2011 to 2015. The optimal number of latent classes and polynomial shapes were determined by comprehensively evaluating the Bayesian Information Criterion (BIC), Akaike Information Criterion (AIC), the proportion of the sample in each class, and clinical interpretability. This step aimed to classify the population into discrete latent classes with different SBP evolutionary characteristics, which served as the core exposure variable for subsequent analyses.

Baseline characteristics in 2011 were described across the identified SBP trajectory groups. Normally distributed continuous variables, non-normally distributed continuous variables, and categorical variables were expressed as mean ± standard deviation (SD), median with interquartile range (IQR), and frequencies with percentages [n (%)], respectively. Differences among groups were evaluated using one-way analysis of variance (ANOVA), the Kruskal-Wallis test, or the Chi-square test, as appropriate based on the data distribution characteristics.

A counterfactual-based causal mediation analysis was conducted to explore the long-term impact of SBP trajectories on the 2018 medical economic burden through the 2015 functional decline (measured by the FI). We decomposed and calculated the following effect indicators: Total Effect (TE), Natural Direct Effect (NDE), and Natural Indirect Effect (NIE). The proportion mediated (NIE/TE × 100%) was also calculated to quantify the contribution of functional decline. All effect estimates and their 95% confidence intervals (CIs) were computed using a non-parametric bootstrapping method with 5000 resamples to ensure the robustness of the statistical inference. Furthermore, multiple mediation models were constructed for sensitivity analyses.

Furthermore, given the close pathophysiological relationship between blood pressure dynamics and body weight fluctuations, we conducted a rigorous sensitivity analysis to isolate the specific effect of blood pressure. Following the identical GBTM procedure used for SBP, we identified longitudinal Body Mass Index (BMI) trajectories from 2011 to 2015. These BMI trajectories were subsequently introduced into the mediation framework as an alternative exposure to verify whether the mediating role of frailty was independent of longitudinal weight changes.

All statistical analyses were conducted using R software. A two-sided *P* < 0.05 was considered statistically significant.

## 3 Result

### 3.1 Result of Group-Based Trajectory Model (GBTM)

This study systematically evaluated systolic blood pressure (SBP) trajectory models encompassing varying numbers of latent classes (ranging from 1 to 5) and different polynomial degrees, specifically comparing first-order linear (Fig. 2) and second-order quadratic (Fig. 3) specifications (Table 1). Based on a comprehensive consideration of model goodness-of-fit (AIC and BIC values), model parsimony, the proportion of the sample in each class, and clinical interpretability, a three-class first-order (linear) trajectory model (Fig. 2c) was ultimately selected as the optimal fitting solution.

**Fig. 2:**
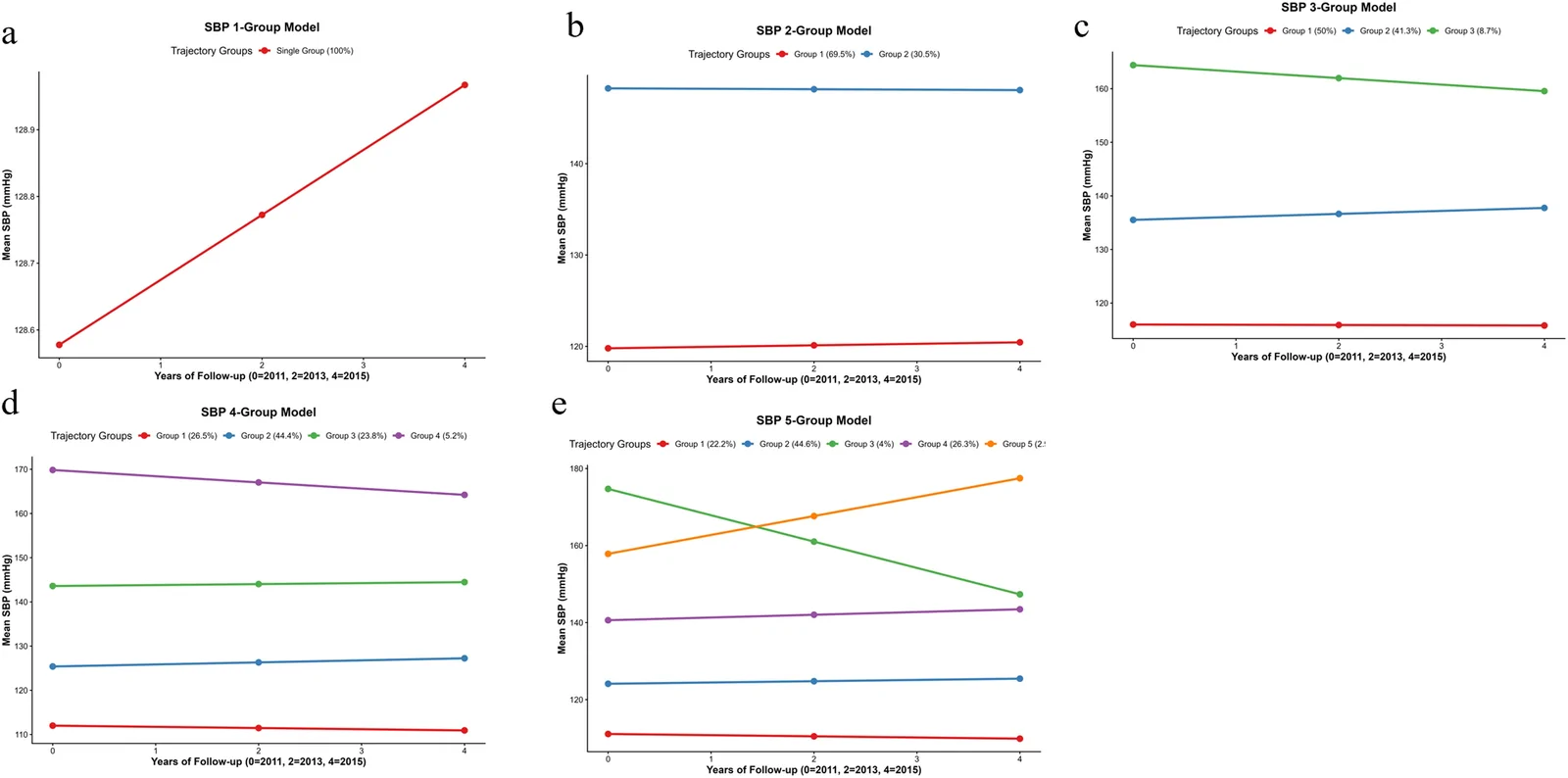
Linear group-based trajectory modeling of systolic blood pressure (SBP) from 2011 to 2015. Panels (a) through (e) illustrate the estimated SBP evolutionary trajectories for models specifying 1–5 latent classes using a first-order (linear) polynomial function. Panel (c) represents the optimal three-class linear model ultimately selected for the primary analysis, comprising the Low-stable (red line, 50.0%), Moderate-increasing (blue line, 41.3%), and High-decreasing (green line, 8.7%) groups.

**Fig. 3:**
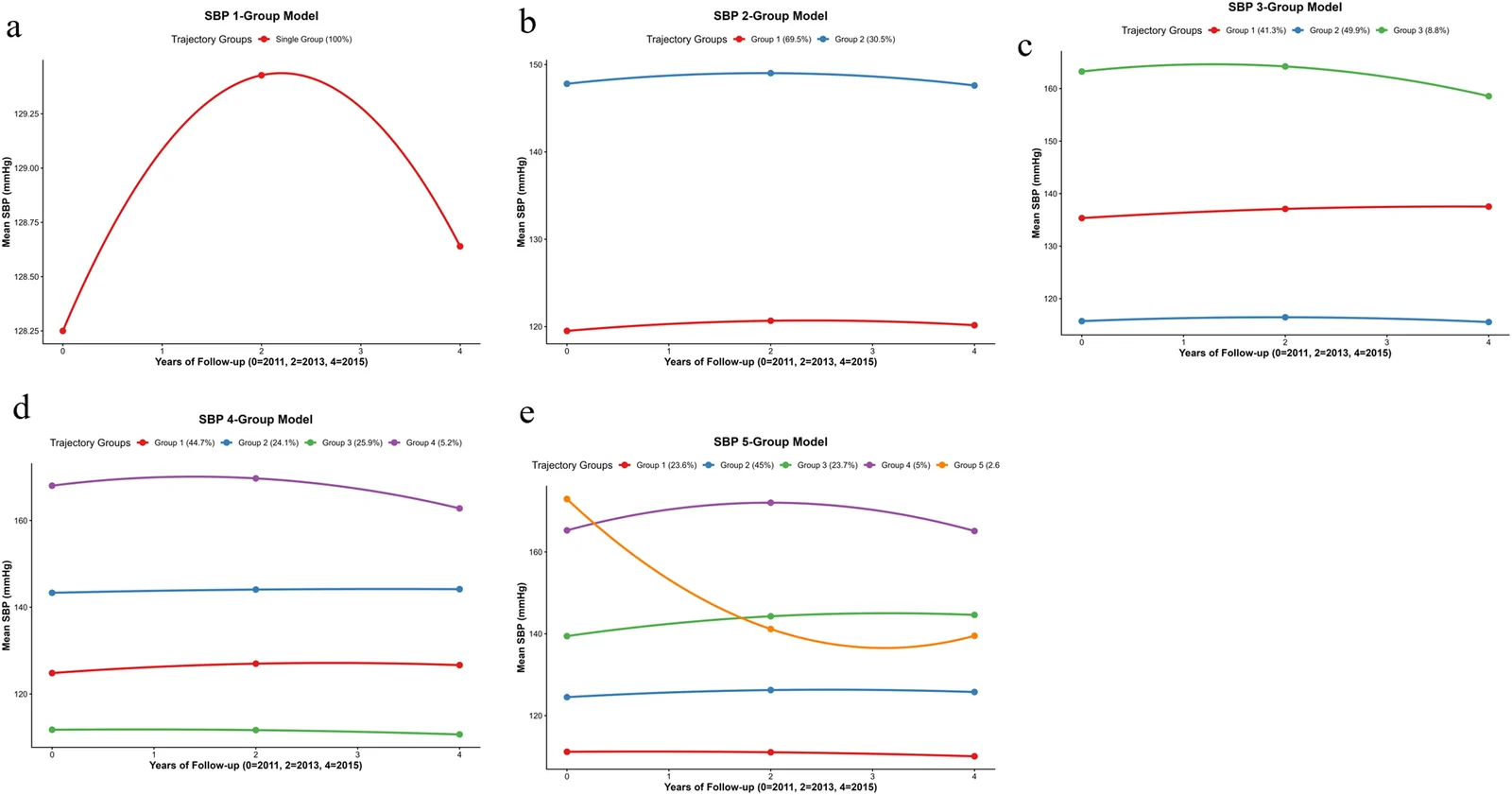
Quadratic group-based trajectory modeling of systolic blood pressure (SBP) from 2011 to 2015. Panels (a) through (e) illustrate the estimated SBP evolutionary trajectories for models specifying 1–5 latent classes using a second-order (quadratic) polynomial function. This quadratic specification is presented for comparison with the optimal linear models. Panel (c) displays the alternative three-class quadratic specification (Class 1: 41.3%; Class 2: 49.9%; Class 3: 8.8%).

**Table 1:** Fit statistics for group-based trajectory models of systolic blood pressure.

Groups	AIC	BIC	Min Prop	Min AvePP
**Linear**				
1	105839.3	105858.2	1	1
2	102939.6	102977.4	0.3051	0.8900
3	102116.2	102172.8	0.0873	0.8552
4	101944.5	102,020	0.0518	0.7584
5	101784.9	101879.3	0.0288	0.7634
**Quadratic**				
1	105834.9	105,860	1	1
2	102,932	102982.4	0.3051	0.8902
3	102103.4	102178.9	0.0878	0.856
4	101931.5	102032.2	0.0523	0.7578
5	101757.1	101882.9	0.026	0.7692

AIC, Akaike information criterion; BIC, Bayesian information criterion; Min Prop, minimum proportion of the trajectory group; Min AvePP, minimum average posterior probability. The selected optimal model (3-group linear) is highlighted in bold.

Utilizing the longitudinal measurement data obtained from 2011 to 2015, the selected linear model successfully classified the study participants (*N* = 3996) into three distinct SBP evolution trajectories. Specifically, Class 1 comprised 50.0% of the total sample, characterized by SBP levels that remained persistently low and relatively stable over the long term, and was designated as the “Low-stable” trajectory. Class 2 accounted for 41.3% of the sample, exhibiting moderate baseline SBP levels followed by a mild increasing trend over time, and was defined as the “Moderate-increasing” trajectory. Class 3, consisting of 8.7% of the participants, manifested elevated baseline SBP but a pronounced declining trend during the follow-up period, and was identified as the “High-decreasing” trajectory. For comparison, the estimated trajectories using the quadratic specification are presented in Fig. 3. In subsequent multivariable regression and causal mediation analyses, the “Low-stable” trajectory from the optimal linear model was uniformly designated as the reference category.

### 3.2 Baseline characteristics of the study population

A total of 3996 respondents were included in this study. Based on the SBP trajectory model, the Low-stable (Group 1), Moderate-increasing (Group 2), and High-decreasing (Group 3) groups accounted for 50.0%, 41.3%, and 8.7% of the study population, respectively. Baseline characteristic analysis revealed substantial heterogeneity across the three trajectory groups regarding several sociodemographic features, clinical indicators, and economic parameters (Table 2). Specifically, individuals in Group 3 exhibited more pronounced sociodemographic vulnerabilities, characterized by advanced age, lower educational attainment, and a higher proportion of non-married status (all *P* ≤ 0.004). Furthermore, the FI score in this group was the highest among the three categories [0.16 *(IQR*: 0.09, 0.26), *P* < 0.001]. In terms of economic dimensions, although Group 1 recorded the highest absolute annual total expenditure, Group 3 demonstrated the highest healthcare expenditure share (0.12 *(IQR*: 0.03, 0.33), *P* = 0.001), suggesting a more prominent relative healthcare expenditure burden within this subgroup.

**Table 2:** Baseline characteristics of participants by systolic blood pressure trajectory groups (*N* = 3996).

Variable	Overall (*N* = 3996)	Group 1 (*N* = 1997)	Group 2 (*N* = 1650)	Group 3 (*N* = 349)	p-value
Age	58 (8)	56 (8)	59 (8)	62 (8)	<0.001
Gender					0.2
Male	2035 (51%)	989 (50%)	866 (52%)	180 (52%)	
Female	1961 (49%)	1008 (50%)	784 (48%)	169 (48%)	
Education Level					0.004
Illiterate/Below primary school	1657 (41%)	788 (39%)	704 (43%)	165 (47%)	
Primary School	970 (24%)	473 (24%)	405 (25%)	92 (26%)	
Middle School	941 (24%)	493 (25%)	381 (23%)	67 (19%)	
High School/Above	428 (11%)	243 (12%)	160 (9.7%)	25 (7.2%)	
Marital Status					<0.001
Married	3669 (92%)	1879 (94%)	1495 (91%)	295 (85%)	
Not married	327 (8.2%)	118 (5.9%)	155 (9.4%)	54 (15%)	
Hukou					0.3
Agricultural	3386 (85%)	1706 (85%)	1381 (84%)	299 (86%)	
Non-agricultural	586 (15%)	281 (14%)	259 (16%)	46 (13%)	
Other	24 (0.6%)	10 (0.5%)	10 (0.6%)	4 (1.1%)	
Dyslipidemia	310 (7.8%)	111 (5.6%)	156 (9.5%)	43 (12%)	<0.001
Diabetes or High Blood Sugar	187 (4.7%)	72 (3.6%)	95 (5.8%)	20 (5.7%)	0.004
Smoking Status	1648 (41%)	788 (39%)	701 (42%)	159 (46%)	0.046
Alcohol Drinking Status					0.027
Drink but less than once a month	334 (8.4%)	181 (9.1%)	135 (8.2%)	18 (5.2%)	
Drink more than once a month	1079 (27%)	505 (25%)	474 (29%)	100 (29%)	
None of these	2583 (65%)	1311 (66%)	1041 (63%)	231 (66%)	
Body Mass Index (kg/m²)	23.8 (3.9)	23.1 (3.5)	24.4 (4.3)	24.8 (3.6)	<0.001
Frailty Index	0.12 (0.06, 0.20)	0.10 (0.05, 0.19)	0.13 (0.06, 0.21)	0.16 (0.09, 0.26)	<0.001
Baseline SBP (mmHg)	128 (20)	115 (11)	136 (14)	165 (17)	<0.001
Annual Medical Expenditure in 2018	2000 (500, 5000)	2000 (500, 5000)	2000 (500, 5000)	2000 (500, 5000)	0.7
Annual Total Expenditure in 2018	20,400 (10,620, 38,240)	21,650 (11,424, 40,730)	19,830 (10,084, 36,400)	16,820 (9,280, 32,000)	<0.001
Medical Expenditure Share in 2018	0.09 (0.03, 0.26)	0.09 (0.02, 0.23)	0.10 (0.03, 0.28)	0.12 (0.03, 0.33)	0.001

Stratified analyses targeting frailty severity and healthcare expenditures further elucidated the co-occurrence and distributional characteristics of these variables (s S4 and 5). When stratified by frailty level, frail individuals (FI ≥ 0.25) were accompanied by a higher prevalence of metabolic comorbidities, with the rates of dyslipidemia and diabetes being significantly elevated compared to the non-frail group (both *P* < 0.001). Concurrently, baseline SBP levels varied significantly across different frailty strata (*P* < 0.001), wherein higher frailty levels were associated with elevated SBP. Additionally, increased frailty severity was significantly correlated with an escalation in direct medical expenditures [2450 vs. 1300 CNY, *P* < 0.001] and a reduction in total expenditures [16,470 vs. 21,580 CNY, *P* < 0.001], consequently driving up the overall healthcare expenditure share (P < 0.001).

When a healthcare expenditure share exceeding 0.40 was utilized as the threshold for a high CHE risk (accounting for 15.5% of the study population), the high-burden group was characterized by advanced age and intensified frailty severity (both *P* < 0.001). Regarding the SBP indicators, the baseline SBP levels were significantly higher in the high-burden group compared to its counterpart (130 vs. 128 mmHg, *P* = 0.014). Analysis of expenditure structures confirmed that the absolute medical expenditure of the high-burden group was tenfold higher than that of the low-burden group [10,000 vs. 1000 CNY, *P* < 0.001], whereas no statistically significant difference was observed in annual total expenditures between the two groups *(P* = 0.8). These cross-sectional descriptive findings preliminarily suggest that specific adverse SBP patterns (e.g., the High-decreasing trajectory) and elevated frailty states may be potentially associated with a significantly increased medical economic burden among the middle-aged and older population.

### 3.3 Mediating role of frailty between SBP trajectories and Catastrophic Health Expenditure (CHE)

Utilizing a causal mediation analysis based on the counterfactual framework, this study evaluated the mediating pathway of frailty (measured by the Frailty Index [FI] in 2015) between SBP trajectories (2011–2015) and the subsequent risk of Catastrophic Health Expenditure (CHE) in 2018. The overarching architecture of this mediation analysis, along with the core regression pathways, is visually summarized in Fig. 4, with detailed model parameters presented in Table 3.

**Fig. 4:**
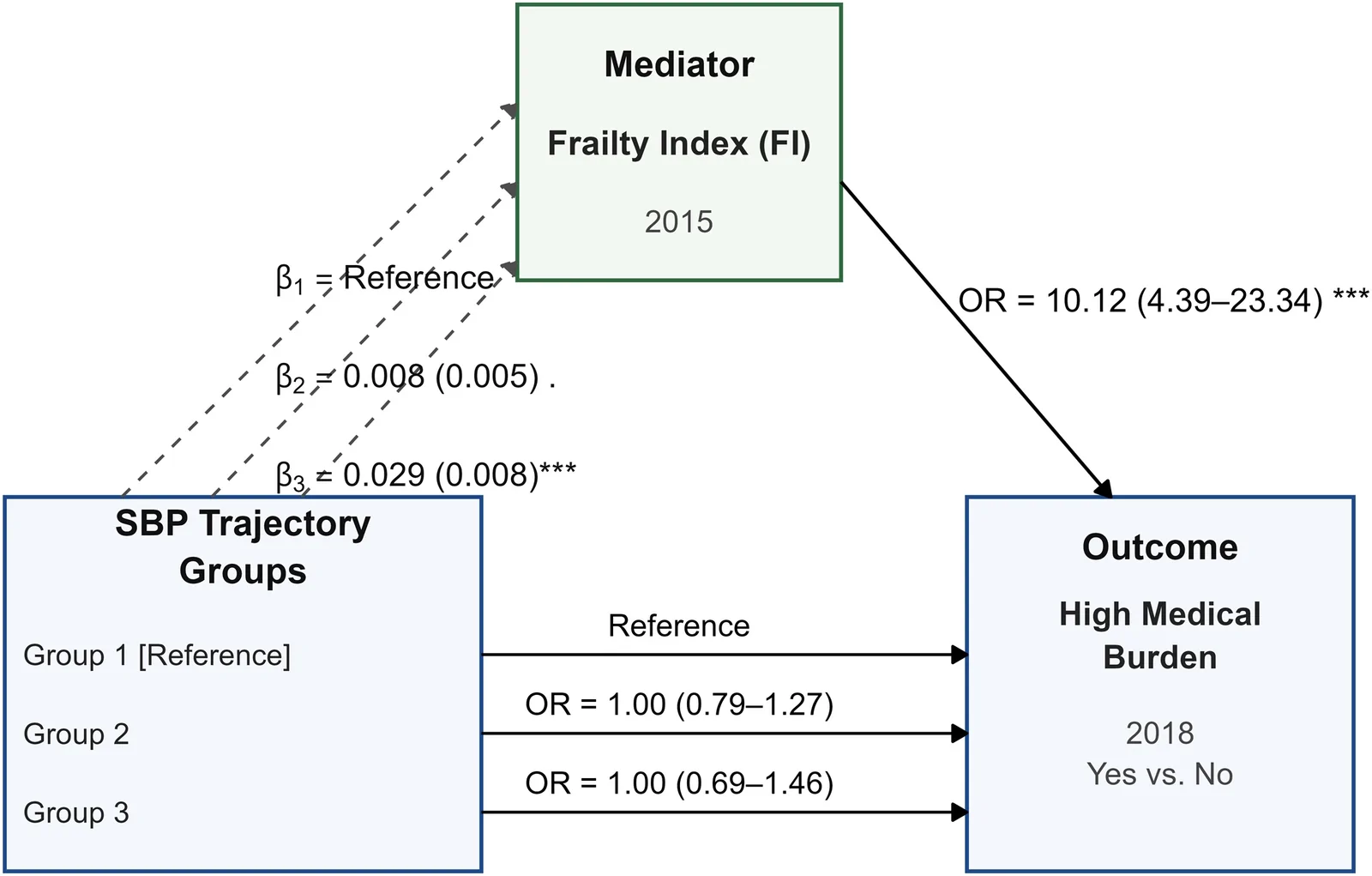
Path diagram of the mediating effect of the Frailty Index on the association between SBP trajectories and CHE. Dashed arrows indicate linear regression coefficients (*β*[SE]) from the mediator model; solid arrows indicate Odds Ratios (OR [95% CI]) from the outcome model. Group 1 (Low-stable) served as the reference category. All models were adjusted for baseline covariates, including age, gender, education, residence, marital status, dyslipidemia, diabetes or high blood sugar, smoking, and alcohol drinking status. (*** *P* < 0.001, ** *P* < 0.01, * *P* < 0.05, † *P* < 0.10). Statistical Significance: *** *P* < 0.001, ** *P* < 0.01, * P<0.05.

**Table 3:** Regression analysis for the mediation effects of Frailty Index on the association between SBP trajectories and Catastrophic Health Expenditure (CHE).

Variables	Model 1: Mediator model (DV: Frailty Index in 2015)	Model 2: Outcome Model (DV: CHE in 2018, Logistic Regression)
	β (SE)	OR (95% CI)
**SBP Trajectory Groups**		
Group 2 (vs Group 1)	0.008 (0.005)	1.00 (0.79−1.27)
Group 3 (vs Group 1)	0.029 (0.008) ***	1.00 (0.69−1.46)
**Mediator**		
Frailty Index (FI) score	–	10.12 (4.39−23.34) ***

Notes: **Abbreviations:** SBP, Systolic Blood Pressure; DV, Dependent Variable; CHE, Catastrophic Health Expenditure; FI, Frailty Index; β, Beta coefficient; SE, Standard Error; CI, Confidence Interval. **Model 1** is a multivariable linear regression model estimating the effect of SBP trajectories on the 2015 Frailty Index. **Model 2** is a multivariable logistic regression model estimating the effects of SBP trajectories and the 2015 Frailty Index on the risk of 2018 CHE. All models were adjusted for baseline covariates including age, gender, and residence status.

**Statistical Significance:** *** *p* < 0.001, ** *p* < 0.01, * *p* < 0.05, † *p* < 0.10 (marginal significance).

In the mediator model (linear regression), after adjusting for baseline covariates, the “High-decreasing” trajectory (Group 3) was significantly associated with higher FI scores in 2015 (*β* = 0.029, *SE* = 0.008, *P* = 0.0003) compared with the “Low-stable” trajectory (Group 1), while the effect for Group 2 was marginally significant (*β* = 0.008, *SE* = 0.005, *P* = 0.093). In the outcome model (logistic regression), the mediator FI exhibited a significant positive association with the risk of CHE in 2018 (*OR* = 10.12, *95% CI*: 4.39−23.34, *P* < 0.001). In this model, the direct predictive effects of Group 2 and Group 3 on the risk of CHE were no longer statistically significant.

Mediation analysis yielded different estimates of the indirect association through frailty across SBP trajectory comparisons (Table 4). For the “High-decreasing” trajectory (Group 3 vs. Group 1), the average causal mediation effect (ACME) was significant (ACME = 0.00835, *95% CI*: 0.00310−0.01000, *P* < 0.001), suggesting a potential mediating role of frailty in the association between this trajectory and subsequent CHE. Notably, the natural direct effect (ADE) and the total effect were not statistically significant (*P* > 0.05). Although the estimated indirect effect was significant, this pattern does not establish complete mediation or confirm a causal pathway. For the “Moderate-increasing” trajectory (Group 2 vs. Group 1), the ACME was marginally significant (ACME = 0.00229, *P* = 0.09), while the direct and total effects were non-significant. Given the non-significance of the total effect, the proportion mediated is not reported, as it does not satisfy the conventional requirements for substantive interpretation.

**Table 4:** Mediation analysis of the potential intermediate role of the Frailty Index in the association between SBP trajectories and CHE in 2018.

Mediation Paths	Estimate	95% CI	p-value
**Group 2 vs. Group 1**			
ACME (Indirect Effect)	0.0023	[−0.0004, 0.0100]	0.090
ADE (Direct Effect)	0.0005	[−0.0266, 0.0300]	0.930
Total Effect	0.0028	[−0.0242, 0.0300]	
**Group 3 vs. Group 1**			
ACME (Indirect Effect)	0.0084	[0.0031, 0.0100]	<0.001***
ADE (Direct Effect)	0.0001	[−0.0445, 0.0500]	0.970
Total Effect	0.0084	[−0.0374, 0.0600]	0.710

Notes: Abbreviations: ACME, Average Causal Mediation Effect, is the conventional model-based term used in mediation analysis and should not be interpreted as proof of causality in this observational study. ADE, Average Direct Effect; CI, Confidence Interval. Estimates represent the change in the probability of experiencing CHE in 2018. Group 1 (Low-stable trajectory) served as the reference category. Nonparametric bootstrap confidence intervals with the percentile method; simulations = 5000; sample size = 3,996. Statistical Significance: *** *P* < 0.001, **P < 0.01, * *P* < 0.05.

### 3.4 Sensitivity analysis

#### 3.4.1 Alternative exposure: BMI trajectories

To test whether our primary conclusions were robust to other metabolic risk patterns, we replaced SBP with 2011–2015 Body Mass Index (BMI) trajectories as an alternative exposure. Based on the GBTM evaluation criteria (Supplementary Table 6), three distinct groups were selected as the optimal trajectory classification (Fig. 5; comprehensive selection process in Supplementary Fig. 1).

**Fig. 5:**
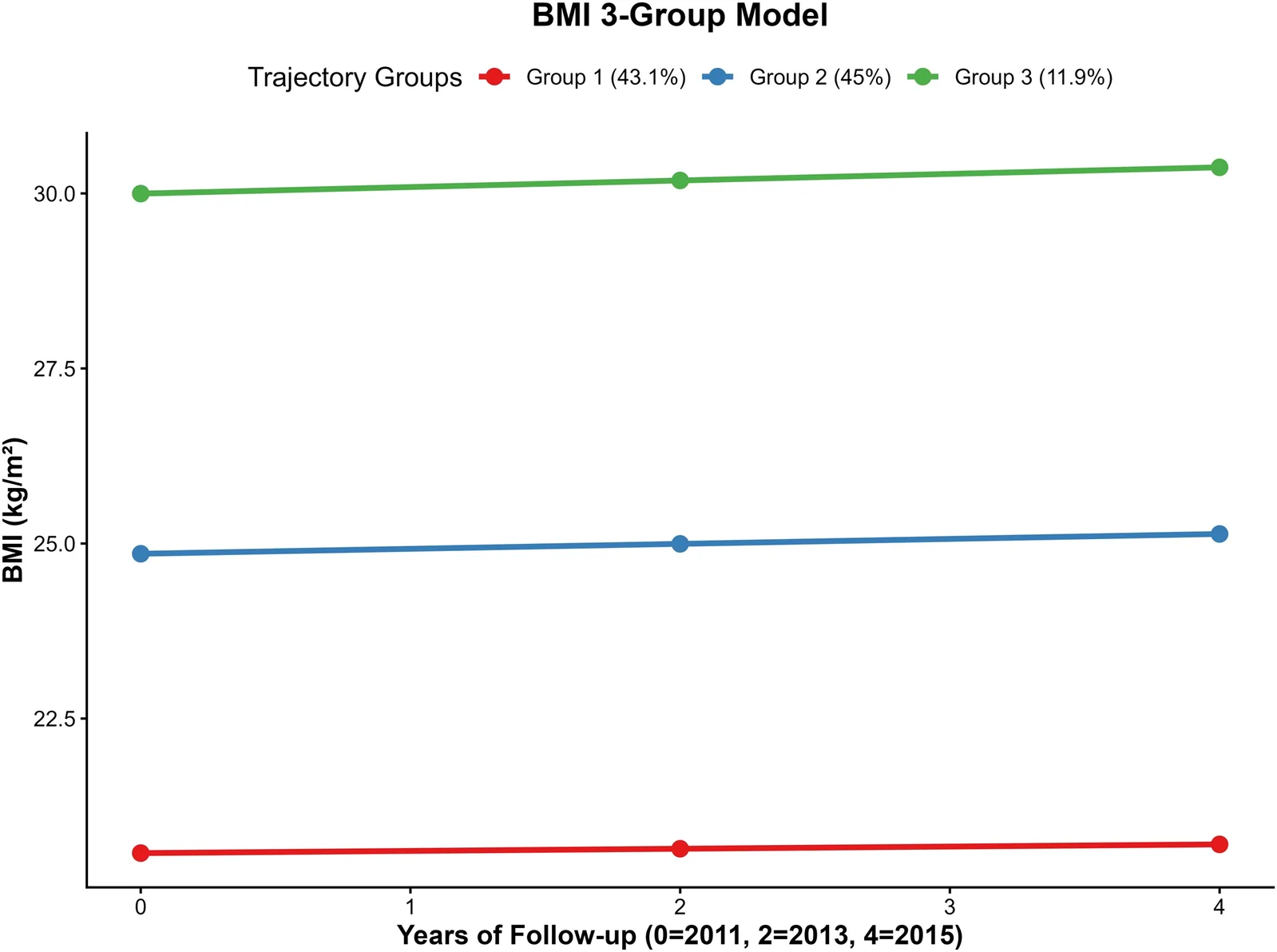
Optimal three-group trajectory model for Body Mass Index (BMI) from 2011 to 2015. Notes: The figure displays the optimal 3-group linear trajectory model for BMI identified using Group-Based Trajectory Modeling (GBTM) for the sensitivity analysis. The x-axis represents the years of follow-up (0 = 2011, 2 = 2013, 4 = 2015), and the y-axis represents the BMI level (kg/m²). The study population was classified into three distinct trajectories: Group 1 (red line, 43.1%), Group 2 (blue line, 45.0%), and Group 3 (green line, 11.9%).

Mediation analyses were then re-constructed (Table 5). Compared with BMI Group 1, Group 2 showed no significant associations with frailty or medical burden. However, Group 3 was significantly associated with higher FI scores in 2015 (*β* = 0.019, P = 0.003). Given that FI strongly predicted CHE in 2018 (OR = 10.04, P < 0.001), adjusting for FI rendered the direct predictive effect of Group 3 completely non-significant (OR = 1.02, 95% CI: 0.76−1.37).

**Table 5:** Regression analysis for the mediation effects of the Frailty Index on the association between BMI trajectories and CHE in 2018.

Variables	Model 1: Mediator model (DV: Frailty Index in 2015) β (SE)	Model 2: Outcome Model (DV: CHE in 2018, Logistic Regression) OR (95% CI)
**BMI Trajectory Groups**		
Group 2 (vs Group 1)	−0.003 (0.004)	0.92 (0.76−1.11)
Group 3 (vs Group 1)	0.019 (0.006) **	1.02 (0.76−1.37)
**Mediator**		
Frailty Index (FI) score	–	10.04 (5.24−19.21) ***

Notes: Abbreviations: BMI, Body Mass Index; DV, Dependent Variable; FI, Frailty Index; OR, Odds Ratio; CI, Confidence Interval; SE, Standard Error. Model 1 is a multivariable linear regression model estimating the effect of BMI trajectories on the 2015 Frailty Index. Model 2 is a multivariable logistic regression model estimating the effects of BMI trajectories and the 2015 Frailty Index on the risk of CHE in 2018. All models were adjusted for baseline covariates including age, gender, education, residence, marital status, dyslipidemia, diabetes or high blood sugar, smoking, and alcohol drinking status.

Statistical Significance: *** *P* < 0.001, ** *P* < 0.01, * *P* < 0.05.

Effect decomposition (Supplementary Table 7) further evaluated this potential pathway. the estimated indirect effect through frailty was statistically significant (*ACME* = 0.00569, *P* = 0.003), whereas the direct and total effects were not statistically significant. This pattern was consistent with a potential indirect association through frailty.

#### 3.4.2 Alternative outcome definitions

To further validate our results from the outcome perspective, we re-conducted the mediation analyses using the original medical burden (a continuous variable) and its log-transformed value [log(medical burden + 1)] as outcome variables (Supplementary Tables 8 and 9). The log transformation aimed to address the right-skewed distribution and zero-inflation issues commonly encountered in medical expenditure data.

When using the untransformed continuous healthcare expenditure share, the results were highly consistent with the primary analysis. In the mediator model, the “High-decreasing” trajectory (Group 3) still exhibited significantly higher frailty levels. The outcome model demonstrated a significant positive association between the 2015 FI score and the 2018 medical burden (*β* = 0.261, SE = 0.036, *P* < 0.001). Effect decomposition (Supplementary Table 9) further supported the mediating role of functional decline: for Group 3 vs. Group 1, a significant ACME was observed (ACME = 0.00765, 95% CI: 0.00303−0.01000, *P* = 0.0012), while the direct and total effects did not reach statistical significance. For Group 2 vs. Group 1, the ACME was only marginally significant (ACME = 0.002127, *P* = 0.089).

Using the log-transformed medical burden (Supplementary Tables 10 and 11) yielded identical conclusions. After adjusting for the same covariates, the FI score remained significantly positively correlated with log [medical share + 1] (*β* = 0.208, SE = 0.027, *P* < 0.001). Mediation effect decomposition revealed that the indirect effect of Group 3 on log[medical share + 1] via the 2015 FI remained significant (*ACME* = 0.00608, *95% CI:* 0.00244−0.01000, *P* < 0.001), while both direct and total effects were non-significant. These sensitivity analyses confirm that regardless of how the outcome variable (categorical or continuous) or the exposure variable (SBP or BMI) is defined, the pathway through which trajectory patterns impact the medical economic burden via functional decline possesses excellent robustness.

## 4 Discussion

Utilizing longitudinal cohort data from the China Health and Retirement Longitudinal Study (CHARLS), this study investigated the interrelationships among systolic blood pressure (SBP) trajectories, frailty, and healthcare economic burden through mediation analysis. Our findings indicate that middle-aged and older individuals exhibiting a “High-decreasing” SBP trajectory had higher levels of frailty at the intermediate assessment. Concurrently, a higher Frailty Index (FI) score was independently associated with a greater likelihood of incurring CHE. Mediation analysis further suggested that frailty may represent a potential intermediate factor in the association between the “High-decreasing” SBP trajectory and subsequent CHE. In summary, this study suggests that frailty may serve as a potential intermediate link association between adverse SBP trajectories and subsequent household CHE.

The present study revealed that the "High-decreasing" SBP trajectory was associated with greater frailty among the middle-aged and older population. This finding is broadly consistent with recent evidence from large-scale longitudinal cohorts, including CHARLS, which suggests that adverse anomalous long-term blood pressure trajectories and visit-to-visit variability can accelerate health deficit accumulation [18,22,35], thereby increasing frailty risk [[18], [19], [20], [21], [22]]. Pathophysiologically, this observed association may stem from cumulative vascular damage associated with earlier elevated blood pressure and changes in health status reflected by subsequent blood pressure decline. A higher cumulative blood pressure burden during the early phase of the trajectory may be associated with target-organ damage and neurocognitive decline [35]. Related research on blood pressure dysregulation also suggests potential links with autonomic dysfunction and reduced physiological complexity, although these mechanisms were not directly assessed in this study [18,36,37]. The ensuing endothelial injury and vascular remodeling may contribute to increased arterial stiffness, potentially compromising the maintenance of adequate tissue perfusion [38,39].

Concurrently, in the face of a stiffened vascular network, a systemic blood pressure collapse precipitates chronic hypoperfusion in the brain and skeletal muscles. Prolonged disturbances in microcirculation and energy metabolism may, in turn, contribute to reduced physiological reserve and frailty [22,35,40]. The subsequent spontaneous decline in blood pressure is likely not an indication of clinical improvement, but rather represents an ominous end-stage manifestation of multi-system failure [41,42]. From a deeper micro-pathological perspective, long-term hemodynamic impairment is not merely a physical insult; it synergistically intertwines with and has the potential to exacerbate metabolic derangements such as insulin resistance, and potentially associated with accelerated ageing at the epigenetic level [16,17,21,43]. Therefore, the depletion of physiological homeostatic reserve and the decline in stress tolerance among individuals with a "High-decreasing" SBP trajectory may help explain their higher levels of frailty [44,45]. This interpretation can also be situated within the healthy ageing framework, which emphasizes maintaining positive health attributes and function alongside assessing disease and vulnerability. Intrinsic capacity (IC), defined by the WHO as the composite of an individual’s physical and mental capacities, provides a complementary perspective to frailty assessment [46]. Recent longitudinal evidence indicates that blood pressure variability has been associated with IC decline [47]. Although BPV and SBP trajectories characterize different aspects of blood pressure dynamics and should not be treated as equivalent, this evidence highlights the importance of considering functional capacities when examining long-term blood pressure changes. As IC was not assessed in the present study, future research should investigate whether adverse SBP trajectories are associated with IC decline and whether such decline is related to subsequent healthcare economic burden.

The potential implications of adverse long-term BP trajectories may extend beyond clinical and physiological outcomes to healthcare utilization and household finances. In addition to acute cardiovascular and cerebrovascular events, sustained healthcare needs associated with frailty may contribute to this burden. Addressing this issue, our mediation analysis based on CHARLS longitudinal data suggests that frailty assessed in 2015 was associated with an increased likelihood of CHE in 2018. The analysis further suggested a potential indirect association between the “High-decreasing” SBP trajectory and subsequent CHE through frailty. By incorporating BP trajectories, frailty, and subsequent economic outcomes into a common analytical framework, our findings support frailty as a potential intermediate factor linking long-term blood pressure changes to household financial burden.

This finding broadens our understanding of the healthcare economic burden associated with cardiovascular risk factors: in addition to acute events such as myocardial infarction and stroke; sustained healthcare needs accompanying frailty may represent another potential source of economic burden. Evidence from multiple large-scale longitudinal studies indicates that the accumulation of chronic conditions is not the only factor associated with increased expenditures; frailty and accompanying functional limitations are also associated with higher healthcare cost [[48], [49], [50]]. Upon entering this stage, patients' healthcare utilization patterns tend to exhibit a marked shift: on the one hand, explicit medical costs surge dramatically due to high-frequency outpatient visits and unplanned emergency hospitalizations, accounting for over 75% of excess costs [51,52]; on the other hand, implicit expenditures manifest through secondary functional impairments, lifelong dependence on expensive professional care, age-friendly home modifications, and productivity losses incurred by family caregiving [53,54]. Furthermore, when further compounded by chronic comorbidities, the risk of household financial hardship may increase [55]—a phenomenon that may extend even into middle age [56].

Evidence from other chronic disease settings also supports the association between frailty and healthcare resource use [57]. In heart failure, secondary frailty not only doubles the frequency of emergency department visits but also skyrockets hospitalization expenses to 2.5 times those of non-frail counterparts [[58], [59], [60]]; whereas in diabetes follow-up cohorts, the accumulation of frailty stands as the core factor mediating exorbitant intensive care and institutional care fees [61,62]. This logic is equally applicable to chronic kidney disease (CKD) and intensive care unit (ICU) settings; evidence indicates that frailty is not only an independent predictor of surging resource consumption in CKD patients but also is strongly linked to ICU patients' sustained dependence on long-term medical services post-discharge [63,64]. More critically, within the evolutionary cascade of cardiometabolic diseases, once the primary chronic disease burden overlaps with secondary frailty, the risk of household financial collapse is exponentially amplified [55,57]. Together, these lines of evidence further support the mediating role of frailty in long-term chronic disease progression and household economic burden. However, unlike previous studies that merely treated frailty as a parallel risk factor, the present study provides empirical evidence suggesting through mediation analysis that frailty serves as a crucial indirect pathway linking adverse BP trajectories to household CHE. This quantitative finding helps elucidate the internal pathway from chronic disease evolution to economic catastrophe, thereby filling an empirical gap in this field.

Even more critically, the present study indicates that as individual frailty intensifies, the sharp escalation of healthcare expenditures coexists with the contraction of total household consumption, and was associated with a higher likelihood of CHE. This suggests that the healthcare consumption associated with frailty may potentially exert a notable medical "crowding-out effect" at the household level. To cope with the exorbitant costs of disability care, households are forced to curtail basic subsistence consumption, such as food and housing, and even sacrifice long-term human capital investments in education [65,66]. Cohort studies and cross-lagged panel models suggest that deteriorating health and financial hardship may be bidirectionally associated [67,68]. This intractable loop of "functional decline-medical impoverishment-accelerated health deterioration" ultimately acts as a profound mechanism that locks vulnerable households into an insurmountable long-term poverty trap [69].

Based on these insights, the present study highlights the need to carefully re-evaluate conventional stringent antihypertensive protocols. For frail individuals exhibiting a 'High-decreasing' trajectory, strictly adhering to standard blood pressure targets yields marginal cardiovascular benefits. Instead, such intensive interventions may escalate the risks of target organ hypoperfusion, falls, and fractures, potentially exacerbating functional impairment [70,71]. Consequently, future cardiovascular management should integrate disease control with assessment of frailty and functional capacities, with the broader goal of preserving IC and functional ability. Based on these insights, the present study highlights the potential value of considering individual frailty alongside conventional blood pressure evaluation in middle-aged and older adults. However, because our study is observational and did not directly evaluate clinical interventions, whether incorporating routine frailty screening or adjusting antihypertensive strategies can actively alter economic trajectories requires further investigation through dedicated clinical trials [72,73].

The primary strength of this study lies in the utilization of the national CHARLS cohort to dynamically characterize long-term SBP trajectories, effectively bridging cardiovascular clinical features with health economics and overcoming the limitations of single-time-point assessments.

However, several limitations should be acknowledged. First, the observational design precludes strict causal inference, and residual confounding and reverse associations may have influenced the findings. Second, the 2015 frailty assessment temporally overlaps with the end of the 2011–2015 trajectory construction. This limits confirmation that the BP trajectories preceded frailty. Accordingly, the mediation analysis should be interpreted as an exploration of potential indirect associations rather than validation of a causal mediation pathway. Third, this study adopted a complete-case analysis approach by excluding participants with missing longitudinal data. This may introduce selection bias, as our final analytical sample likely represents a relatively 'healthier' survivor cohort. Consequently, the direction and magnitude of the resulting selection bias cannot be determined with certainty, and the generalizability of our findings to more vulnerable populations may be limited. Fourth, economic outcomes were assessed in 2018 and may not fully reflect subsequent changes in health insurance policies, healthcare utilization, and household expenditure patterns. Fifth, self-reported expenditures are susceptible to recall bias. Sixth, the lack of ambulatory blood pressure monitoring (ABPM) and detailed medication regimens limits the assessment of short-term blood pressure fluctuations and treatment-related changes, as well as our ability to distinguish among different explanations for declining blood pressure. Finally, generalizability to different populations and healthcare financing systems requires external validation evaluation.

## 5 Conclusions

In conclusion, sing data from a nationally representative longitudinal cohort, this study found that middle-aged and older adults with a “High-decreasing” systolic blood pressure trajectory had higher levels of frailty. Mediation analysis suggested a potential mediating role of frailty in the association between the High-decreasing SBP trajectory and subsequent household CHE. These findings suggest the potential value of looking beyond isolated blood pressure targets and recognizing the interplay between long-term hemodynamic patterns and physiological reserve. Future prospective and interventional studies are warranted to explore whether integrating functional assessments into cardiovascular management could help mitigate adverse socioeconomic consequences in this vulnerable population.

## CRediT authorship contribution statement

Weili Liu: Conceptualization, Methodology, Formal analysis, Writing - Original draft, Writing - Review & Editing. Zhonghua Yin: Data curation, Formal analysis, Software, Visualization, Writing - Original draft. Ke Li: Conceptualization, Supervision, Writing - Review & Editing, Project administration, Funding acquisition.

Weili Liu and Zhonghua Yin contributed equally to this work and share first authorship. Ke Li and Weili Liu contributed equally to this work and share corresponding authorship.

## Funding statement

The author(s) declare that financial support was received for the research and/or publication of this article. The project was supported by the 2024 Sichuan Provincial Science and Technology Achievement Transfer and Transformation Guidance Program (No. 2024ZHCG0018) and the 2025 Sichuan Provincial Key Research and Development Program (No. 2025YFHZ0318).

## Human ethics and consent to participate declarations

The study was a secondary analysis of publicly available data from the China Health and Retirement Longitudinal Study (CHARLS). The original CHARLS study protocol was approved by the Institutional Review Board (IRB) of Peking University, and all participants provided written informed consent. Therefore, no further ethics approval was required for this secondary analysis.

## Ethical approval

This study is a secondary analysis of data from the China Health and Retirement Longitudinal Study (CHARLS). The original CHARLS study was approved by the Biomedical Ethics Review Committee of Peking University (IRB00001052-11015). All participants provided written informed consent.

## Declaration of Generative AI and AI-assisted technologies in the writing process

During the preparation and revision of this manuscript, the authors used Gemini to assist with language polishing. The authors reviewed and edited all content and take full responsibility for the content of the publication. No generative AI tools were used to create or modify figures, images, or artwork.

## Data availability

The datasets analyzed during the current study are available in the China Health and Retirement Longitudinal Study (CHARLS) repository, and can be accessed through the official website (https://charls.pku.edu.cn/).

## Declaration of competing interest

The authors declare no conflicts of interest.

## Figures and Tables

**Figure:**