# Supplementary material for: Mediating role of frailty in the association between systolic blood pressure trajectories and catastrophic health expenditure among middle-aged and older adults: A longitudinal cohort study
Source: J Nutr Health Aging. 2026 Oct 30;30(12):101021. doi: 10.1016/j.jnha.2026.101021 (PMC13737738; doi:10.1016/j.jnha.2026.101021)
Supplement: Supplementary file 1 [file mmc1.docx]

Supplemental Table 1. Characteristics of included and excluded participants across available study waves

| **Variable** | **Overall (N=14,462)** | **Included (N=3,996)** | **Excluded (N=10,466)** | **P value** |
| --- | --- | --- | --- | --- |
| Age, years | 59.4 (9.7) | 58.1 (8.0) | 59.9 (10.3) | <0.001 |
| Mean SBP, 2011 (mmHg) | 129.3 (20.6) | 128.2 (19.9) | 131.1 (21.9) | <0.001 |
| Mean SBP, 2013 (mmHg) | 130.1 (20.7) | 129.4 (20.3) | 131.3 (21.5) | <0.001 |
| Mean SBP, 2015 (mmHg) | 129.1 (20.1) | 128.6 (19.8) | 130.0 (20.6) | 0.011 |
| BMI, 2015 (kg/m²) | 23.6 (3.9) | 23.8 (3.9) | 23.5 (3.8) | 0.002 |
| Frailty Index, 2015 | 0.11 (0.06, 0.20) | 0.12 (0.06, 0.20) | 0.10 (0.06, 0.20) | 0.217 |
| Annual medical expenditure, 2018 | 2,000.00 (500.00, 5,000.00) | 2,000.00 (500.00, 5,000.00) | 2,000.00 (450.00, 5,000.00) | 0.670 |
| Annual total expenditure, 2018 | 21,924.00 (10,930.00, 41,300.00) | 20,400.00 (10,620.00, 38,240.00) | 22,996.00 (11,140.00, 43,366.00) | <0.001 |
| Medical expenditure share, 2018 | 0.09 (0.02, 0.25) | 0.09 (0.03, 0.26) | 0.09 (0.02, 0.25) | 0.022 |
| **Gender** |  |  |  | **0.329** |
| Female | 7,185 (49.7%) | 1,961 (49.1%) | 5,224 (50.0%) |  |
| Male | 7,267 (50.3%) | 2,035 (50.9%) | 5,232 (50.0%) |  |
| **Education level** |  |  |  | **<0.001** |
| Illiterate | 6,529 (45.4%) | 1,657 (41.5%) | 4,872 (47.0%) |  |
| Primary School | 3,048 (21.2%) | 970 (24.3%) | 2,078 (20.0%) |  |
| Middle | 2,971 (20.7%) | 941 (23.5%) | 2,030 (19.6%) |  |
| High School/Above | 1,819 (12.7%) | 428 (10.7%) | 1,391 (13.4%) |  |
| **Marital status** |  |  |  | **<0.001** |
| Married | 12,631 (87.3%) | 3,669 (91.8%) | 8,962 (85.6%) |  |
| Not married | 1,830 (12.7%) | 327 (8.2%) | 1,503 (14.4%) |  |
| **Hukou** |  |  |  | **<0.001** |
| Agricultural | 11,426 (79.0%) | 3,386 (84.7%) | 8,040 (76.9%) |  |
| Non-agricultural | 2,933 (20.3%) | 586 (14.7%) | 2,347 (22.4%) |  |
| Other | 98 (0.7%) | 24 (0.6%) | 74 (0.7%) |  |
| **Dyslipidemia** |  |  |  | **0.280** |
| No | 13,180 (92.6%) | 3,686 (92.2%) | 9,494 (92.8%) |  |
| Yes | 1,049 (7.4%) | 310 (7.8%) | 739 (7.2%) |  |
| **Diabetes or high blood sugar** |  |  |  | **0.832** |
| No | 13,694 (95.2%) | 3,809 (95.3%) | 9,885 (95.2%) |  |
| Yes | 683 (4.8%) | 187 (4.7%) | 496 (4.8%) |  |
| **Smoking status** |  |  |  | **0.664** |
| No | 8,530 (59.0%) | 2,348 (58.8%) | 6,182 (59.2%) |  |
| Yes | 5,916 (41.0%) | 1,648 (41.2%) | 4,268 (40.8%) |  |
| **Alcohol drinking status** |  |  |  | **0.648** |
| Drink but less than once a month | 1,184 (8.2%) | 334 (8.4%) | 850 (8.1%) |  |
| Drink more than once a month | 3,836 (26.6%) | 1,079 (27.0%) | 2,757 (26.4%) |  |
| None of these | 9,421 (65.2%) | 2,583 (64.6%) | 6,838 (65.5%) |  |
| **CHE (>40% expenditure share), 2018** |  |  |  | **0.932** |
| No | 8,641 (84.4%) | 3,376 (84.5%) | 5,265 (84.4%) |  |
| Yes | 1,592 (15.6%) | 620 (15.5%) | 972 (15.6%) |  |

Values are mean (SD), median (Q1, Q3), or n (%). P values compare observed data only: Welch two-sample t-test, Kruskal-Wallis test, or Pearson chi-square test with 10,000 Monte Carlo replicates.

| Supplemental Table 2. The 27 items used to construct the frailty index | |  |
| --- | --- | --- |
| Description of the item | Cut-off value |  |
| Self-reported physician diagnosed hypertension | Yes = 1, No = 0 |  |
| Self-reported physician diagnosed diabetes | Yes = 1, No = 0 |  |
| Self-reported physician diagnosed arthritis | Yes = 1, No = 0 |  |
| Self-reported physician diagnosed chronic lung disease | Yes = 1, No = 0 |  |
| Self-reported physician diagnosed any emotional, nervous, or psychiatric problems | Yes = 1, No = 0 |  |
| Self-reported physician diagnosed memory-related disease | Yes = 1, No = 0 |  |
| Self-reported vision problems | Yes = 1, No = 0 in the CHARLS; Poor or fair = 1, excellent, very good, or good = 0 in the ELSA and HRS |  |
| Self-reported hearing problems | Yes = 1, No = 0 in the CHARLS; Poor or fair = 1, excellent, very good, or good = 0 in the ELSA and HRS |  |
|  |  |  |
| Self-reported general health status | Very poor or poor = 1,  Very good, good, or fair = 0 |  |
| Difficulty with dressing | Yes = 1, No = 0 |  |
| Difficulty with bathing or showering | Yes = 1, No = 0 |  |
| Difficulty with eating | Yes = 1, No = 0 |  |
| Difficulty with getting in and out of bed | Yes = 1, No = 0 |  |
| Difficulty with using the toilet | Yes = 1, No = 0 |  |
| Difficulty with managing money | Yes = 1, No = 0 |  |
| Difficulty with taking medication | Yes = 1, No = 0 |  |
| Difficulty with shopping for groceries | Yes = 1, No = 0 |  |
| Difficulty with preparing meals | Yes = 1, No = 0 |  |
| Mobility: difficulty with walking 100 yards or one block | Yes = 1, No = 0 |  |
| Mobility: difficulty with getting up from a chair after sitting for long periods | Yes = 1, No = 0 |  |
| Mobility: difficulty with climbing several flights of stairs without resting | Yes = 1, No = 0 |  |
| Mobility: difficulty with lifting or carrying weights over 10 pounds/jins | Yes = 1, No = 0 |  |
| Mobility: difficulty with picking up a coin from the table | Yes = 1, No = 0 |  |
| Mobility: difficulty with stooping, kneeling, or crouching | Yes = 1, No = 0 |  |
| Mobility: difficulty with reaching arms above shoulder level | Yes = 1, No = 0 |  |
| Depression: CESD-10 questionnaire | CESD-10 >10 = 1, ≤10 = 0 in the CHARLS; CESD-8 ≥ 4 = 1, <4 = 0 in the ELSA and HRS |  |
| Cognition: (memory test score + orientation test score) / 14 | Continuous variable, ranging from 0 to 1 |  |

**Supplemental Table 3**. Definitions of household expenditure measures and catastrophic health expenditure

| Variables |  |
| --- | --- |
| Total annual household expenditure | (1) Weekly expenditures (multiplied by 52), including spending on food, dining out, tobacco, and alcohol; (2) Monthly expenditures (multiplied by 12), including communication, utilities (water and electricity), fuel, domestic services, transportation, daily necessities, and entertainment; (3) Annual expenditures, including clothing, travel, heating, household appliances, education, medical care, health products, personal care, vehicle purchase and maintenance, property management fees, taxes, and donations. |
| Annual medical expenditure | The household’s total medical expenses over the past year, including direct medical costs and indirect costs such as transportation, nutrition, and caregiver services. |
| Proportion of medical expenditure | $\frac{Annual medical expenditure}{Total annual household expenditureCapacity to pay (Total annual expenditure - Food expendi)ture)}\times100\%$ |

**Supplemental** Table 4. Characteristics of participants according to Frailty Index category

| **Variable** | **Overall**  (N=3996) | **Robust/Fit**  (N=1869) | **Pre-frail**  (N=1463) | **Frail**  (N=664) | **p-value**^2^ |
| --- | --- | --- | --- | --- | --- |
| **Age** | 58 (8) | 57 (8) | 59 (8) | 61 (8) | <0.001 |
| **Gender** |  |  |  |  | <0.001 |
| Male | 2,035 (51%) | 1,112 (59%) | 685 (47%) | 238 (36%) |  |
| Female | 1,961 (49%) | 757 (41%) | 778 (53%) | 426 (64%) |  |
| **Education Level** |  |  |  |  | <0.001 |
| Illiterate | 1,657 (41%) | 609 (33%) | 654 (45%) | 394 (59%) |  |
| Primary School | 970 (24%) | 440 (24%) | 377 (26%) | 153 (23%) |  |
| Middle School | 941 (24%) | 535 (29%) | 314 (21%) | 92 (14%) |  |
| High School/Above | 428 (11%) | 285 (15%) | 118 (8.1%) | 25 (3.8%) |  |
| **Marital Status** |  |  |  |  | <0.001 |
| Married | 3,669 (92%) | 1,755 (94%) | 1,327 (91%) | 587 (88%) |  |
| Not married | 327 (8.2%) | 114 (6.1%) | 136 (9.3%) | 77 (12%) |  |
| **Hukou** |  |  |  |  | <0.001 |
| Agricultural | 3,386 (85%) | 1,534 (82%) | 1,266 (87%) | 586 (88%) |  |
| Non-agricultural | 586 (15%) | 323 (17%) | 187 (13%) | 76 (11%) |  |
| Other | 24 (0.6%) | 12 (0.6%) | 10 (0.7%) | 2 (0.3%) |  |
| **Dyslipidemia** | 310 (7.8%) | 111 (5.9%) | 132 (9.0%) | 67 (10%) | <0.001 |
| **Diabetes or High Blood Sugar** | 187 (4.7%) | 41 (2.2%) | 89 (6.1%) | 57 (8.6%) | <0.001 |
| **Smoking Status** | 1,648 (41%) | 854 (46%) | 569 (39%) | 225 (34%) | <0.001 |
| **Alcohol Drinking Status** |  |  |  |  | <0.001 |
| Drink but less than once a month | 334 (8.4%) | 178 (9.5%) | 108 (7.4%) | 48 (7.2%) |  |
| Drink more than once a month | 1,079 (27%) | 591 (32%) | 375 (26%) | 113 (17%) |  |
| None of these | 2,583 (65%) | 1,100 (59%) | 980 (67%) | 503 (76%) |  |
| **Body Mass Index (kg/m²)** | 23.8 (3.9) | 23.6 (3.7) | 23.9 (4.0) | 23.8 (4.3) | 0.11 |
| **SBP in 2011 (mmHg)** | 128 (20) | 126 (18) | 129 (20) | 131 (23) | <0.001 |
| **SBP in 2013 (mmHg)** | 129 (20) | 128 (19) | 130 (20) | 132 (23) | <0.001 |
| **SBP in 2015 (mmHg)** | 129 (20) | 127 (19) | 129 (20) | 132 (21) | <0.001 |
| **Annual Medical Expenditure in 2018, CNY** | 2,000 (500, 5,000) | 1,300 (300, 4,000) | 2,000 (550, 5,000) | 2,450 (600, 5,500) | <0.001 |
| **Annual Total Expenditure in 2018, CNY** | 20,400 (10,620, 38,240) | 21,580 (11,400, 41,194) | 20,424 (10,600, 37,820) | 16,470 (8,729, 32,168) | <0.001 |
| **Medical Expenditure Share in 2018** | 0.09 (0.03, 0.26) | 0.07 (0.01, 0.19) | 0.11 (0.03, 0.29) | 0.17 (0.05, 0.40) | <0.001 |

**Supplemental** Table 5. Characteristics of participants according to CHE status in 2018.

| **Variable** | **Overall**  (N=3996) | **No catastrophic expenditure**  (N=3376) | **Catastrophic expenditure**  (N=620) | **p-value**^2^ |
| --- | --- | --- | --- | --- |
| **Age** | 58 (8) | 58 (8) | 60 (8) | <0.001 |
| **Gender** |  |  |  | 0.9 |
| Male | 2,035 (51%) | 1,717 (51%) | 318 (51%) |  |
| Female | 1,961 (49%) | 1,659 (49%) | 302 (49%) |  |
| **Education Level** |  |  |  | <0.001 |
| Illiterate/Primary | 1,657 (41%) | 1,366 (40%) | 291 (47%) |  |
| Primary School | 970 (24%) | 796 (24%) | 174 (28%) |  |
| Middle School | 941 (24%) | 824 (24%) | 117 (19%) |  |
| High School/Above | 428 (11%) | 390 (12%) | 38 (6.1%) |  |
| **Marital Status** |  |  |  | 0.2 |
| Married | 3,669 (92%) | 3,092 (92%) | 577 (93%) |  |
| Not married | 327 (8.2%) | 284 (8.4%) | 43 (6.9%) |  |
| **Hukou** |  |  |  | 0.003 |
| Agricultural | 3,386 (85%) | 2,834 (84%) | 552 (89%) |  |
| Non-agricultural | 586 (15%) | 524 (16%) | 62 (10%) |  |
| Other | 24 (0.6%) | 18 (0.5%) | 6 (1.0%) |  |
| **Dyslipidemia** | 310 (7.8%) | 254 (7.5%) | 56 (9.0%) | 0.2 |
| **Diabetes or High Blood Sugar** | 187 (4.7%) | 152 (4.5%) | 35 (5.6%) | 0.2 |
| **Smoking Status** | 1,648 (41%) | 1,375 (41%) | 273 (44%) | 0.13 |
| **Alcohol Drinking Status** |  |  |  | 0.13 |
| Drink but less than once a month | 334 (8.4%) | 290 (8.6%) | 44 (7.1%) |  |
| Drink more than once a month | 1,079 (27%) | 925 (27%) | 154 (25%) |  |
| None of these | 2,583 (65%) | 2,161 (64%) | 422 (68%) |  |
| **Body Mass Index (kg/m²)** | 23.8 (3.9) | 23.8 (4.0) | 23.6 (3.7) | 0.2 |
| **Frailty Index** | 0.12 (0.06, 0.20) | 0.10 (0.06, 0.20) | 0.17 (0.09, 0.28) | <0.001 |
| **SBP in 2011 (mmHg)** | 128 (20) | 128 (20) | 130 (21) | 0.014 |
| **SBP in 2013 (mmHg)** | 129 (20) | 129 (20) | 131 (21) | 0.077 |
| **SBP in 2015 (mmHg)** | 129 (20) | 128 (19) | 131 (22) | <0.001 |
| **Annual Medical Expenditure in 2018, CNY** | 2,000 (500, 5,000) | 1,000 (300, 3,000) | 10,000 (5,000, 27,000) | <0.001 |
| **Annual Total Expenditure in 2018, CNY** | 20,400 (10,620, 38,240) | 20,490 (10,871, 37,950) | 19,398 (9,540, 43,976) | 0.8 |
| **Medical Expenditure Share in 2018** | 0.09 (0.03, 0.26) | 0.07 (0.02, 0.16) | 0.58 (0.48, 0.76) | <0.001 |

**Supplementary** Table 6. GBTM Fitted Parameters for BMI Trajectories (2011–2015)

| **Linear** | | | | |
| --- | --- | --- | --- | --- |
| **Groups** | **AIC** | **BIC** | **Min Prop** | **Min AvePP** |
| 1 | 66993.61 | 67012.53 | 1 | 1 |
| 2 | 62052.2 | 62090.04 | 0.3497 | 0.9344 |
| 3 | 59763.34 | 59820.1 | 0.1188 | 0.9162 |
| 4 | 58645.58 | 58721.25 | 0.0501 | 0.8838 |
| 5 | 57375.54 | 57470.13 | 0.0015 | 0.8982 |
| **Quadratic** | | | | |
| **Groups** | **AIC** | **BIC** | **Min Prop** | **Min AvePP** |
| 1 | 66990.43 | 67015.65 | 1 | 1 |
| 2 | 62044.5 | 62094.95 | 0.3500 | 0.9341 |
| 3 | 59746.73 | 59822.41 | 0.1181 | 0.9161 |
| 4 | 58609.58 | 58710.48 | 0.0484 | 0.8846 |
| 5 | 56982.99 | 57109.11 | 0.0015 | 0.9008 |

Notes: Abbreviations: GBTM, Group-Based Trajectory Modeling; BMI, Body Mass Index; AIC, Akaike Information Criterion; BIC, Bayesian Information Criterion; Min Prop, Minimum Proportion of the sample assigned to a trajectory group; Min AvePP, Minimum Average Posterior Probability of assignment across groups. The optimal model was selected based on a comprehensive consideration of model parsimony (lower AIC and BIC values), adequate sample size in each group (Min Prop > 0.05), and good model classification accuracy (Min AvePP > 0.70). Consequently, a 3-group linear model was selected for the BMI trajectory analysis. Although the four-group linear model yielded slightly lower AIC and BIC values, its smallest trajectory group accounted for approximately 5% of participants, and the additional group did not provide substantial clinical distinctiveness or trajectory separation. Therefore, the three-group linear model was ultimately selected based on a comprehensive balance of model parsimony, classification quality, group size, and clinical interpretability

**Supplementary** Table 7. Mediation analysis of the association between BMI trajectories and CHE in 2018 through the Frailty Index.

| **GROUP 2** | **Condition** | **Estimate** | **95% CI** | **p-value** |
| --- | --- | --- | --- | --- |
| ACME | Control | -0.00104 | [-0.00348, 0.00] | 0.39 |
|  | Treated | -0.00098 | [-0.00329, 0.00] | 0.39 |
|  | Average | -0.00101 | [-0.00336, 0.00] | 0.39 |
| ADE | Control | -0.01106 | [-0.03559, 0.01] | 0.38 |
|  | Treated | -0.01100 | [-0.03544, 0.01] | 0.38 |
|  | Average | -0.01103 | [-0.03549, 0.01] | 0.38 |
| Total Effect | - | -0.01204 | [-0.03647, 0.01] | 0.33 |
| GROUP 3 | **Condition** | **Estimate** | **95% CI** | **p-value** |
| ACME | Control | 0.00565 | [0.00188, 0.01] | 0.0032 ** |
|  | Treated | 0.00573 | [0.00196, 0.01] | 0.0032 ** |
|  | Average | 0.00569 | [0.00191, 0.01] | 0.0032 ** |
| ADE | Control | 0.00278 | [-0.03638, 0.04] | 0.8812 |
|  | Treated | 0.00286 | [-0.03761, 0.04] | 0.8812 |
|  | Average | 0.00282 | [-0.03687, 0.04] | 0.8812 |
| Total Effect | - | 0.00851 | [-0.03158, 0.05] | 0.6748 |

Notes: Abbreviations: ACME, Average Causal Mediation Effect (Indirect Effect); ADE, Average Direct Effect. Nonparametric bootstrap confidence intervals with the percentile method; simulations=5000. Statistical Significance: *** p<0.001, ** p<0.01, * p<0.05.

**Supplemental** Table 8. Regression analysis of the potential mediating role of the Frailty Index in the association between SBP trajectories and healthcare expenditure share in 2018

| **Variables** | **Model 1: Mediator model (DV: Frailty Index in 2015)** | **Model 2: Outcome Model (DV: Medical Share in 2018)** |
| --- | --- | --- |
|  | β (SE) | β (SE) |
| **SBP Trajectory Groups** |  |  |
| Group 2 (vs Group 1) | 0.008 (0.005) | 0.0039 (0.0089) |
| Group 3 (vs Group 1) | 0.029 (0.008) *** | 0.0047 (0.0148) |
| **Mediator** |  |  |
| Frailty Index (FI) score | - | 0.2615 (0.0359) *** |

Notes: Model 1 is the same mediator model shown in Table 7. Model 2 is a multivariable linear regression model estimating the effects of SBP trajectories and the 2015 Frailty Index on medical share in 2018. All models were adjusted for the same covariates as Table 7. Statistical Significance: *** p<0.001, ** p<0.01, * p<0.05

**Supplemental** Table 9. Mediation analysis of SBP trajectories on Medical Share in 2018 through the Frailty Index.

| **GROUP 2** | **Condition** | **Estimate** | **95% CI** | **p-value** |
| --- | --- | --- | --- | --- |
|  | ACME | 0.002127 | [-0.000356, 0.00] | 0.089 |
|  | ADE | 0.003854 | [-0.013769, 0.02] | 0.670 |
|  | Total Effect | 0.005981 | [-0.011624, 0.02] | 0.504 |
| **GROUP 3** | **Condition** | **Estimate** | **95% CI** | **p-value** |
|  | ACME | 0.00765 | [0.00303, 0.01] | 0.0012 ** |
|  | ADE | 0.00466 | [-0.02567, 0.04] | 0.7636 |
|  | Total Effect | 0.01231 | [-0.01760, 0.04] | 0.4292 |

Notes: Model 1 is the mediator model reported in Table 3 of the main manuscript. Model 2 is a multivariable linear regression model estimating the associations of SBP trajectory groups and the 2015 Frailty Index with healthcare expenditure share in 2018. Both models were adjusted for baseline covariates including age, gender, educational level, hukou status, marital status, dyslipidemia, diabetes or high blood sugar, smoking status, and alcohol drinking status." simulations=5000; sample size=3996. Statistical Significance: *** p<0.001, ** p<0.01, * p<0.05

**Supplemental** Table 10. Regression analysis of the potential mediating role of the Frailty Index in the association between SBP trajectories and log-transformed healthcare expenditure share in 2018

| **Variables** | **Model 1: Mediator model (DV: Frailty Index in 2015)** | **Model 2: Outcome Model (DV: log[medical_share2018+1])** |
| --- | --- | --- |
|  | β (SE) | β (SE) |
| **SBP Trajectory Groups** |  |  |
| Group 2 (vs Group 1) | 0.008 (0.005) . | 0.0032 (0.0067) |
| Group 3 (vs Group 1) | 0.029 (0.008) *** | 0.0049 (0.0112) |
| **Mediator** |  |  |
| Frailty Index (FI) score | - | 0.2077 (0.0272) *** |

Notes: Model 1 is the mediator model reported in Table 3 of the main manuscript. Model 2 is a multivariable linear regression model estimating the associations of SBP trajectory groups and the 2015 Frailty Index with healthcare expenditure share in 2018. Both models were adjusted for baseline covariates including age, gender, educational level, hukou status, marital status, dyslipidemia, diabetes or high blood sugar, smoking status, and alcohol drinking status.

Statistical Significance: *** p<0.001, ** p<0.01, * p<0.05

**Supplemental** Table 11. Mediation analysis of the association between SBP trajectories and log-transformed healthcare expenditure share in 2018 through the Frailty Index

| **GROUP 2** | **Condition** | **Estimate** | **95% CI** | **p-value** |
| --- | --- | --- | --- | --- |
|  | ACME | 0.001689 | [-0.000283, 0.00] | 0.095 |
|  | ADE | 0.003176 | [-0.009780, 0.02] | 0.648 |
|  | Total Effect | 0.004866 | [-0.008459, 0.02] | 0.484 |
| **GROUP 3** | **Condition** | **Estimate** | **95% CI** | **p-value** |
|  | ACME | 0.00608 | [0.00244, 0.01] | <0.001 *** |
|  | ADE | 0.00489 | [-0.01817, 0.03] | 0.68 |
|  | Total Effect | 0.01097 | [-0.01220, 0.03] | 0.34 |

*Notes: Nonparametric bootstrap confidence intervals with the percentile method; simulations=5000; sample size=3996. Statistical Significance: *** p<0.001, ** p<0.01, * p<0.05.*
